# Today’s advanced is tomorrow’s basic

**DOI:** 10.1186/s13089-018-0100-9

**Published:** 2018-08-15

**Authors:** Joe Betcher, Al Majkrzak, Ross Kessler, Nik Theyyunni, Rob Huang

**Affiliations:** 1 Department of Emergency Medicine, Lake Michigan Emergency Specialists, Mercy Health Muskegon, Muskegon, MI USA; 2 Department of Emergency Medicine, Emergency Physicians Medical Group, St. Joseph Mercy Ann Arbor, Ypsilanti, MI USA; 3 Department of Emergency Medicine, University of Michigan, Ann Arbor, MI USA

We have great respect for Dr. Blanco and Dr. Bello’s deep knowledge and extensive experience. However, we must disagree with the desire to postpone care until patients are in the intensive care unit. The idea that a provider should wait for someone else to care for our patients is antithetical to the basic premise of point of care ultrasound. The care a patient receives should be based on their needs, not on what floor of the hospital their bed is on currently.

This study was born out of the long length of stay of the critically ill patients in our emergency department. To address this issue, our emergency department had recently created an ED-ICU hybrid care area called the Emergency Critical Care Center (EC3). Patients may be in the ED or this unit up to 24 h, being treated primarily by an emergency physician. We also note that there may be significant intercountry variation in typical scope of point of care ultrasound use. Advanced echocardiographic measurements are not routinely made by many of the intensivists in our hospital.

We agree that basic echocardiography and pulmonary ultrasound skills form the bedrock of the assessment of ED and ICU patients. Basic bedside cardiac ultrasound and pulmonary ultrasound are routine in the evaluation of many of our patients, and are a milestone within emergency medicine training in the US [1, 2]. We agree that the use of lung ultrasound to evaluate for distribution of A-lines, B-lines, effusions and consolidations can add immensely to the information gained from basic echocardiography. This bears repeating—trainees should be focused on excellence in applying these basic skills. Indeed, outside of this feasibility study we do not routinely train novices in these measurements.

While basic TTE is useful across a broad range of patients, we would anticipate that these more advanced measures would be useful only more selectively. It is as yet not well-studied what percentage of ED or ICU patients would benefit from this kind of evaluation, but we agree it is doubtless that fewer patients would benefit from basic TTE. As you noted, our results show that these measurements are both moderately time consuming and challenging when performed by novice users. We also agree that not every ED physician needs to use these measurements to initiate resuscitation for the complex cardiopulmonary patient.

Obtaining diastolic or VTI parameters can be performed on a case-by-case basis, and may be most useful when comparison imaging including previous comprehensive echocardiography is available. Further we agree it is likely most useful in those practitioners who have spent months or years becoming an expert in it. Finally, we absolutely agree that obtaining the LVOT outflow tract diameter measurements is unnecessary, and only leads to increased chance of error with increased amounts of time needed to perform the scan. However, standard echocardiographic protocols have included this measurement, and we felt it appropriate to include for study. In our clinical practice, when VTI is used, the LVOT diameter is not routinely measured.

It is important not to overextend the meaning of a feasibility study. We believe you took away more or less what the results of this study shows—that these measurements can be challenging and time consuming for novice sonographers—but under some circumstances feasible. Further, work is necessary to understand whether a selective application of these measurements would be useful in patient care.

Finally, we feel that it is important to remember that the scope of ultrasound use is typically determined in a specialty-specific manner. We would be uncomfortable determining the appropriate scope of practice of an intensivist, and are similarly concerned about the suggestion that an intensivist determines what is most appropriate for an emergency physician. Rather than imagining care and research in this area to be siloed as “ED” or “ICU” appropriate, we feel this work should be collaborative across POCUS users of relevant disciplines.
